# Supplementary material for: Temporal-spatial variability of modern climate in the Altai Mountains during 1970-2015
Source: PLoS One. 2020 Mar 17;15(3):e0230196. doi: 10.1371/journal.pone.0230196 (PMC7077806; doi:10.1371/journal.pone.0230196)
Supplement: Attachment — Submitted filename: PONE.docx [file pone.0230196.s002.docx]

The reviewer congratulates the authors for this extensive analysis of “**Temporal-spatial variability of modern climate in the Altai Mountains during 1970-2015**”. This is a noble piece of work and will be very useful for the scientific community. This paper uses station data and the results are mostly based on the statistical analysis. However, there are some minor issues related with the present manuscript to be addressed before its acceptance:

1. Line 78: I think it is species not ‘specie’
2. Provide a better figure 2. The years at the bottom is not clear.
3. Line no 169-170 : please rephrase “Around 21.69 mm of annual 170 mean precipitation was increased from 1970 to 2015”
4. Line 176: “with significant levels:. You need to mention the value
5. Line 190: change ‘trends was’ to ‘trends were’
6. Line 198: please change the heading to “mean annual cycle or temporal evolution of temperature and precipitation”.
7. The tables are missing from the manuscript. Please provide.
8. The elevation dependent temperature depends upon the various factor, such as snow-albedo feedback, nocturnal cloud, change in surface energy balance, aerosol and change in water vapour and fluxes. The authors must look in to these to confirm which mechanism dominates over Altai mountains in deciding the elevation dependent warming.
