# Supplementary material for: Temporal-spatial variability of modern climate in the Altai Mountains during 1970-2015
Source: PLoS One. 2020 Mar 17;15(3):e0230196. doi: 10.1371/journal.pone.0230196 (PMC7077806; doi:10.1371/journal.pone.0230196)
Supplement: Attachment — Submitted filename: Authors Replies to Review Comments.doc [file pone.0230196.s003.doc]

From: zhdl@ms.xjb.ac.cn

**Authors replies to the review comments**

Plos One (PONE-D-19-22736)

Dear Professor Yang Bao:

Thanks very much for taking your and the reviewers’ time to review this manuscript. We really appreciate all your comments and suggestions! Please find our responses in below and my revisions/corrections in the re-submitted files.

Thank you very much for your attention and consideration again.

Sincerely yours

Zhang Dongliang

2019-12-11

**Replies to Editor (thank you!):**

One reviewer raised serious concerns, and thought this manuscript is similar with the paper published in Scientific Reports from the same research team in 2018. Therefore, I would suggest you have to address this concern carefully. Specific attention should also be paid as follows:

**Reply:** Yes, we published the paper in Scientific Reports in 2018. That paper revealed the out-of- phase relationship of precipitation changes at different time-scales (i.e., season, year, multi-decades, centennial and millennial scales). In this study, we systematically investigated the temporal-spatial changes of modern climate in the Altai Mountains during 1970-2015, including temperature and precipitation trends and their changes in different sub-regions, temperature and precipitation trend-elevation relationship and possible reasons.

**Replies to Reviewer 1 (thank you!):**

Line 78: I think it is species not ‘specie’

**Reply:** Modified.

Provide a better figure 2. The years at the bottom is not clear.

**Reply:** Added.

Line 169-170: please rephrase “Around 21.69 mm of annual mean precipitation was increased from 1970 to 2015”.

**Reply:** Done.

This MAP in 2015 increased 21.69 mm comparing with that in 1970.

Line 176: “with significant levels:. You need to mention the value.

**Reply:** Added.

Line 190: change ‘trends was’ to ‘trends were’

**Reply:** Added.

Line 198: please change the heading to “mean annual cycle or temporal evolution of temperature and precipitation”.

**Reply:** Thank you. We changed the heading to “evolution of temperature and precipitation in sub-regions”.

The tables are missing from the manuscript. Please provide.

**Reply:** Added.

The elevation dependent temperature depends upon the various factor, such as snow-albedo feedback, nocturnal cloud, change in surface energy balance, aerosol and change in water vapour and fluxes. The authors must look into these to confirm which mechanism dominates over Altai mountains in deciding the elevation dependent warming.

**Reply:** Done.

This character should result from the lakes (e.g., Wulungu Lake, Zaysan Lake, Teleskoyel Lake and Achit Lake) in low-elevation regions of the Altai Mountains, which significantly alter the surface energy and then influence on regional climate via mediating the fluxes of energy, moisture and momentum [9, 47-48]. In details, during the warm season (summer and autumn), the lakes absorb incoming solar radiation and inhibit upward turbulent heat transport, the humid air has a lower probability of being heated by latent heat released from condensation at low elevations which induce cooling and lower trend in low elevation. During the cold season (spring and winter), the air is warmed and showed a greater trend magnitudes in low elevations due to the heat is released from lakes.

**Replies to Reviewer 2 (thank you!):**

1, For the introduction section, I believe some recent literatures about climate changes over the high-elevation regions, would also be helpful to strengthen the motivation and importance of this manuscript, such as:

**Reply:** We added the several papers you suggested.

2, Line 137, any physical explanations about the largest increasing trend in spring?

**Reply:** Thank you for your question. We investigated the possible causes.

The rising rate of temperature was the quickest in spring, which was attributable to the substantially declined snow cover inferred from satellites in the past 30 years, especially in early spring through summer interval (Zhai and Zhou, 1997; Qian et al., 2011; Huang et al., 2012). Fossil fuel and biofuel emissions of black carbon plus organic matter were mainly responsible for springtime snow cover loss over Eurasia (Flanner et al., 2009).

Huang JP, Guan XD, Ji F. Enhanced cold-season warming in semi-arid regions. Atmospheric Chemistry and Physics, 2012; 12(12): 5391-5398.

3, The three sub-regions used in Fig. 7 should be marked in a map.

**Reply:** Thank you! We showed these sub-regions in Figure 1b.

4, In Fig. 8, which stations are categorized as high-, middle-, low-elevations?
**Reply:** Thank you! We added them in the revised manuscript.

We further analyzed the temperature/precipitation trends in different elevations. The Altai Mountains were divided into three gradients (i.e., >2000 m, 1000-2000 m and <1000 m) (Fig. 8). The stations within >2000 m were Kara-Tyurek and Yalalt. Mugur-Aksy, Kosh-Agach, Olgiy, Khovd and Qinghe were contained elevation between 2000 and 1000 m and the remaining stations were considered elevation <1000 m.

5, About the section of “temperature/precipitation trend-elevation relationship”, first, I am not surprised about no significant relationship between precipitation trends and elevations. But, for the relationship between temperature trends and elevation, usually larger increasing trends over high-elevation regions are more acceptable to the whole community, because of several potential mechanisms: snow albedo, water vapor changes, and aerosols etc. (Pepin et al., 2015). In Fig. 8, 0.037 C/decade, 0.044 C/decade, and 0.039C/decade are not very different, especially how many stations in each category are not given. Therefore, I would recommend the authors use another data (e.g., CRU data) to to repeat this analysis and confirm their results.

**Reply:** Thank you. Your suggestion is very valuable. According to your suggestion, we checked CRU and GPCC data. The study showed that both CRU and GPCC products are able to describe the temporal-spatial variation of precipitation in China. However, the CRU data showed large biases in the Tibetan Plateau and some large-mountain areas including the Tianshan Mountains and the Altai Mountains (Wang and Wang, 2017). The study also showed that GPCC is a better choice compared to CRU for studying the long-term precipitation trend in China (Wang and Wang, 2017). Although GPCC agrees well with observed data, the uncertainty of the satellite rainfall products limits their applicability in mountainous areas (Jin et al., 2016). Therefore, we pay more attention on the observed data in the Altai Mountains.

Wang D, Wang AH. Applicability assessment of GPCC and CRU precipitation products in China during 1901 to 2013. Climatic and Environmental Research, 2017; 22(4): 446-462 (in Chinese).

Jin XL, Shao H, Zhang C, Yan Y. The Applicability Evaluation of Three Satellite Products in Tianshan Mountains. Journal of Natural Resources, 2016; 31(12): 2074-2085 (in Chinese).

6, In the following discussion, the authors tried to use the snow cover to explain the converse relationships between temperature trends and elevations. But, I think seasonal snow cover extents can only be used to explain the climatology, rather than the changes. While, the changes of snow cover and surface albedo feedback will support the elevation dependent warming. Deeper discussion about changes of snow cover and corresponding mechanisms is needed in the revision.
**Reply:** Done.

This character should result from the lakes (e.g., Wulungu Lake, Zaysan Lake, Teleskoyel Lake and Achit Lake) in low-elevation regions of the Altai Mountains, which significantly alter the surface energy and then influence on regional climate via mediating the fluxes of energy, moisture and momentum [9, 47-48]. In details, during the warm season (summer and autumn), the lakes absorb incoming solar radiation and inhibit upward turbulent heat transport, the humid air has a lower probability of being heated by latent heat released from condensation at low elevations which induce cooling and lower trend in low elevation. During the cold season (spring and winter), the air is warmed and showed a greater trend magnitudes in low elevations due to the heat is released from lakes.

7, In the section of “association between atmospheric circulations and climate information”, when using correlations between precipitation changes and large-scale climate variability to explain the precipitation changes, the authors mixed the concepts of inter-annual variations and trends. Meanwhile, AO, NAO, and ENSO are inter-annual scale climate variability, while AMO and PDO are decadal scale climate variability, so they influence precipitation variability on different scales. Therefore, the authors need to clarify which of these two changes (or both) they want to discuss, then discuss the corresponding mechanisms. Moreover, the mechanisms behind influences from the AO, NAO, and AMO are easy to understand through the water vapor transportation through the westerly. While, the authors should provide more details about mechanisms behind influences from PDO and ENSO to the precipitation over the Altai Mountains.
**Reply:** Thank you very much. According to your valuable suggestion, we didn’t discuss the associations between precipitation and driving factors (including AO, NAO, ENSO and so on) because no obvious correlations were found in the revised manuscript. In this study, we pay more attention on the effect of atmospheric circulations on the climate changes of the Altai Mountains.

As shown in Fig. 11, the Altai Mountains were controlled by cyclonic anomalies to their northwest and an anticyclonic anomalies to their southeast. This kind of atmospheric modes blocked the northward cold air and in turn provided favorable environment for an increasing temperature in the Altai Mountains. The water vapor from the North Atlantic, the Mediterranean and Caspian sea was transported to the Altai Mountains via the westerlies (Aizen et al., 2001, 2006). Previous study indicated that the sum of horizontal moisture advection and wind convergence terms has a significant positive contribution to the wetting trend in the northwest region of China (Peng and Zhou, 2017). As shown in Fig. 11, the southwestern wind between the anomaly cyclone and the anomaly anticyclone driven the water vapor flux from the Mediterranean and Caspian sea into the Altai Mountains, which in turn, increases the precipitation. The larger increased precipitation appeared in sub-region 1 and 3 during 1970-2015 due to the effect of windward slope with a significant change in southern Altai Mountains within China [27].

8, In Fig. 11, why the whole period is divided into 1996-2016 and 1960-1995? Any sudden change or shift around 1995/96?
**Reply:** Thank you for your question. Yes, you are right. The mutations of MAT occurred in 1995 and the large atmospheric circulation influenced on the mutations of climate. This result was inferred from the analysis of temperature in the nearby Tianshan Mountains by Xu et al. (2018).

9, Maybe I missed some files, but I did not see Table 1 and Table 2.
**Reply:** We are sorry we missed them in the original manuscript. Now we added the table in the resubmitted manuscript.

10, Caption of Fig. 10, should it be mean annual precipitation?
**Reply:** Done.

**Replies to Reviewer 3 (thank you!):**

1. Lines 35-36: Why does the warming result in the wetting in mid-latitude regions?

**Reply:** Thank you. Sorry, we made a mistaken and modified it. The AR5 of IPCC (2013) pointed out that in the 21st century global warming will further intensify the Earth’s water cycles, making the high-latitude areas even wetter and mid- and low-latitude areas even drier, melting more glaciers and reducing spring snow covers in the Northern Hemisphere.

Intergovernmental Panel on Climate Change Fifth Assessment Report (IPCC AR5). Summary for Policymakers: The Physical Science Basis, Contribution of Working Group I to the IPCC Fifth Assessment Report Climate Change, 2013.

2. Lines 37-38: Revise “the mid-latitude regions” to “Eurasia”.

**Reply:** Done.

3. Where are the tables?

**Reply:** Added.

4. Line 108: AMO and PDO are not atmosphere circulation indices.

**Reply:** Deleted.

5. Lines 107-116: Why do you select these five climate indices, which cannot explain the results in this study as analyzed in the Discussions of this paper?

**Reply:** Thank you for your question. Based on no obvious correlation between five indices and climate changes, we deleted this part. We focused on the analysis of association between atmospheric circulations and climate information.

6. Lines 113-116: Why do you unconventionally divide the seasons?

**Reply:** Modified and reanalyzed data.

Winter, spring, summer and autumn are defined as extending from December to the following February (DJF), March to May (MAM), June to August (JJA) and September to November (SON), respectively.

7. Lines 212-218: It is better to display the sub-regions in Figure 1.

**Reply:** Displayed.

8. Lines 279-281: Figure 10c indicates larger changes in lower elevations than that in the higher elevations.

**Reply:** Modified.

9.Lines 285-299: The correlations reflect the interannual relationships between the indices and precipitation in the Altai Mountains. However, this study focused on the decadal variability of precipitation in the Altai Mountains. Hence, this analysis is improper for this study.

**Reply:** Thank you for your valuable suggestion. We deleted the correlation analysis and paid more attention on association between atmospheric circulations and climate information.

10.Lines 300-315: This study explored the climate changes during 1970-2015, whereas Figure 11 analyzed the circulation changes from 1960 to 2016. Hence, this analysis is also improper for this study.

**Reply:** Thank you. We referenced the study between atmospheric circulations and climate changes during 1960-2016 in the nearby Tianshan Mountains, which was finished by Xu et al. (2018).

11. Lines 285-315: The first paragraph suggests that global circulations have weak relationship with the precipitation. The second paragraph indicates that local circulation might modulate the influence of global circulations on precipitation. However, the detailed mechanism is not proposed in this study.

**Reply:** Thank you. We paid more attention on association between atmospheric circulations and climate information.

The authors suggest that this anticyclone mode blocked the water vapour transport from the west. Why does this weakened water vapour transport not cause a drying trend? Why does the strengthening of Siberian High in winter not cause a cooling in the Altai Mountains?

**Reply:** Thank you. We modified it.

As shown in Fig. 11, the Altai Mountains were controlled by cyclonic anomalies to their northwest and an anticyclonic anomalies to their southeast. This kind of atmospheric modes blocked the northward cold air and in turn provided favorable environment for an increasing temperature in the Altai Mountains.

Figure 11 indicates that the Altai Mountains are controlled by cyclonic anomalies to their northwest and an cyclonic anomalies to their southeast, rather than an anticyclone circulation.

**Reply:** According to your suggestion, we modified it.

As shown in Fig. 11, the Altai Mountains were controlled by cyclonic anomalies to their northwest and an anticyclonic anomalies to their southeast. This kind of atmospheric modes blocked the northward cold air and in turn provided favorable environment for an increasing temperature in the Altai Mountains.

11. Fig. 10: Revise “air temperature” to “precipitation”.
**Reply:** Changed.

**Replies to Reviewer 4 (thank you ! ):**

1. Tables that mentioned in the manuscript are missing.

**Reply:** Added.

2. The trends of annual and seasonal mean temperature/precipitation for the fifteen stations in the Altai Mountains have been investigated and compared with each other. A table containing this information can be added to make readers easier to follow. In the analysis of trend-elevation relationship, which stations are included in the high elevations, middle elevations, and low elevations need to be clarified.

**Reply:** Thank you! We added them.

We further analyzed the temperature/precipitation trends in different elevations. The Altai Mountains were divided into three gradients (i.e., >2000 m, 1000-2000 m and <1000 m) (Fig. 8). The stations within >2000 m were Kara-Tyurek and Yalalt. Mugur-Aksy, Kosh-Agach, Olgiy, Khovd and Qinghe were contained elevation between 2000 and 1000 m and the remaining stations were considered elevation <1000 m.

3. The three subregions are divided based on the three characters of monthly precipitation. For the northern Altai Mountains (subregion 1), the monthly precipitation in Zmeinogorsk station shows two peaks (Fig. 6), but the other five stations in this sub-region all shows one peak. Why is the Zmeinogorsk station included in this subregion? More explanation is needed. At the same time, I have the similar concern about the southern Altai Mountains (subregion 3). This subregion includes Qinghe, Aletai, Fuyun, and Habaha stations. The monthly precipitation of Qinghe station shows one peak, but two peaks can be found in other three stations. The division of sub-regions need more justifications.

**Reply:** Thank you very much for pointing out the mistake. We did not notice it. Based on your suggestion, we redivided the sub-regions.

Fig. 6 showed monthly temperature and monthly precipitation changes of the Altai Mountains during 1970-2015. The highest temperature appears in June-August and the lowest temperature in November-January. The highest temperature (22.97 °C) in summer was recorded in Zajsan and the lowest value (-28.78 °C) in winter was in Kosh-Agach (Fig. 6a). Three features of monthly precipitation are characterized in the Altai Mountains (Fig. 6b). Firstly, the monthly precipitation is unimodal and is mainly concentrated in warm season (summer and autumn) with 55-84%. The related stations are Soloneshnoe, Kyzyl-Ozek, Yailu, Kara-Tyurek and Ust-Coksa. Their MAP is also relatively abundant (average about 649.73 mm). Secondly, the feature of monthly precipitation is also unimodal, but the MAP is relatively low (average about 133.86 mm). The associated stations are Mugur-Aksy, Kosh-Agach within Russia, three stations in Mongolia and Qinghe in China. Thirdly, different to the former two features of precipitation, the distribution of monthly precipitation is bimodal characterized by two peaks at April-September (50-68%) and at November-December (13-21%). The associated stations include Zmeinogorsk in Russia, Habahe, Aletai and Fuyun in China, Semipalatinsk and Zajsan in Kazakhstan.

4. For the atmospheric circulations responsible for the climate variability in the Altai Mountains is not clear. This manuscript stated that the Altai Mountains experienced a rapid warming (line 318, line 22), but it also argued that no rapid warming in the Altai Mountains due to the strengthening of Siberian High in winter (lines 308-309). This is contradictory. Meanwhile, how does the anticyclone mode block less water vapor transported by the westerlies from the North Atlantic Ocean into the Altai? I don’t think the reference 35 talks about it.
**Reply:** Thank you very much. We rewritten them.

Regional precipitation variability in climate trends could be closely related with atmosphere circulation. Xu et al. [9] calculated that differences of geopotential height (shaded) and wind speed (vector) at 500 hPa in the Asian Central Arid Zone including the Altai Mountains through calculating the circulation using NCEP/NCAR reanalysis data (Fig. 11). As shown in Fig. 11, the Altai Mountains are controlled by cyclonic anomalies to their northwest and an anticyclonic anomalies to their southeast. This kind of atmospheric modes blocked the northward cold air and in turn provided favorable environment for an increasing temperature in the Altai Mountains. The water vapor from the North Atlantic, the Mediterranean and Caspian sea is transported to the Altai Mountains via the westerlies (Aizen et al., 2001, 2006). Previous study indicated that the sum of horizontal moisture advection and wind convergence terms has a significant positive contribution to the wetting trend in the northwest region of China (Peng and Zhou, 2017). As shown in Fig. 11, the southwestern wind between the anomaly cyclone and the anomaly anticyclone drives the water vapor flux from the Mediterranean and Caspian sea into the Altai Mountains, which resulted in an increasing precipitation. The larger increased precipitation appeared in sub-region 1 and 3 due to the effect of windward slope with a significant change in the southern Altai Mountains within China [27].

Specific comments:
1. Lines 125: the subtitle “Temperature and precipitation during 1970-2015” is redundant.

**Reply:** Deleted.

2. There is a small figure on the left bottom of figure 1. The description about it needs to be added to the caption of figure 1.

**Reply:** Added.

3. For figure 3, there is a small histogram and ”0.47” on the top of the legend. What does that stand for? Figure 5 has the same problem.

**Reply:** Thank you very much. We modified them. The numbers in a small histogram of Figure 3 and 5 indicate the changeable values of temperature and precipitation.

4. The figure 6 doesn’t have “a” on the temperature (left figure) and “b” on the precipitation (right figure)

**Reply:** Added.

5. For the caption of figure 8, change “annual mean precipitation” to” mean annual precipitation”

**Reply:** Modified.

6. The “seasonal air temperature” needs to be added to the caption of figure 9 and figure 10.

**Reply:** Added.

7. In figure 10, the left string (annual, summer, etc.) covered the data point.

**Reply:** Modified.

5. The Altai Mountains has been divided into 3 subregions (northern, eastern and southern). Showing these subregions in a figure will be clearer. Maybe the division of subregions can be added to the figure 1.

**Reply:** Added.
