# Supplementary material for: Temporal-spatial variability of modern climate in the Altai Mountains during 1970-2015
Source: PLoS One. 2020 Mar 17;15(3):e0230196. doi: 10.1371/journal.pone.0230196 (PMC7077806; doi:10.1371/journal.pone.0230196)
Supplement: Attachment — Submitted filename: Authors Replies to Review Comments2.doc [file pone.0230196.s004.doc]

From: zhdl@ms.xjb.ac.cn

**Authors replies to the review comments**

Plos One (PONE-D-19-22736R1)

Dear Prof. Yang Bao:

Thanks very much for taking your and the reviewers’ time to review this manuscript. We really appreciate all your comments and suggestions! Please find our responses in below and my revisions/corrections in the re-submitted files.

Thank you very much for your attention and consideration again.

Sincerely yours

Zhang Dongliang

2020-2-10

Detailed comments:

1. Lines 27-28: Revise “in the northwest” to “ to the northwest”, “in the southeast” to “ to the southeast”, and “northward” to “southward” (northward air is warm air, also in Line 296).

**Reply:** Done.

2. Lines 30 and 302: “southwestern” to “southwesterly”

**Reply:** Done.

3. Lines 45-46: “leading to put the security of water resources at risk” has syntax error.

**Reply:** Done.

4. Line 48 and other places in this paper: “divergent” is more appropriate than “changeable”.

**Reply:** Done.

5. Line 68: “detailed” to “detailedly”

**Reply:** Done.

6. Line 70: “are” to “is”

**Reply:** Done.

7. Fig. 2e: p>0.01

**Reply:** Modified.

8. Lines 119-123 and Lines 153-157: The comparisons are meaningless because they are not in the same period. The trends would be different even in the same area between different periods.

**Reply:** Deleted.

9. Lines 127-128: “Fig. 2b-d” to “Fig. 2b-e”

**Reply:** Done.

10.Line 161: “Fig. 4b-d” to “Fig. 4b-e”

**Reply:** Done.

11.Line 209: “both” is inappropriate

**Reply:** Deleted.

12. Lines 247-259: The interpretation is not convincing. The lake areas are very small compared with the study area. The effect of the lake on the temperature is local and very weak. In addition, the lakes are freezing up in cold seasons, which would cool rather than warm the air via reflecting solar radiation.

**Reply:** Thank you very much. We neglect this important information about the freezing lakes in winter.

This elevation-dependent temperature depends upon the changes of snow cover and surface albedo feedback in the Altai Mountains [9, 47-48]. In details, the larger snow cover and its stronger albedo feedback in cold season accelerate the transit of upward turbulent heat, the cooling air has a lower probability of being heated by latent heat in high elevation than that in low elevation. In warm season, the decreased snow cover and the weakening surfce albedo can not inhibit the warming air in high elevation, i.e., high-elevation environments experience more rapid changes in temperature than environments at lower elevations [2-5].

13.Fig. 10b: p>0.01

**Reply:** Done.

14. Lines 271-273: Negative trend in lower elevation also indicates precipitation change, the range of which is larger than that in higher elevation.

**Reply:** Modified.

15. Fig. 11: which season? In addition, the differences can be calculated by the authors according the study period rather than modifying from Xu et al. (2018).

**Reply:** Thank you for your question. Fig. 11 shows the annual-scale differences of geopotential height (shaded) and wind speed (vector) at 500 hPa between 1996-2016 and 1960-1995. We referenced the Xu et al. (2018)’s results based on two reasons: firstly, the climate in the Tianshan Moutains and the Altai Mountains within the Central Asia are consistently influenced by the westerlies throughout a year and by the Siberian High in winter; secondly, the destination of this manuscript is from our study about Holocene climate change in the Altai Mountains. We recognized a delayed temperature increase at high elevations during the early Holocene, resulting in more humid conditions at high elevations compared to low elevations (Zhang and Feng, 2018). So we want to investigate the temporal and spatial change of climate in the observed interval. Honestly, we have difficulty in doing that. Thank you very much.

16. Line 315: CRU and GPCC are not satellite-based data.

**Reply:** We deleted the word “satellite”.

17. Lines 100-101 and Lines 318-320: In the Data Section, it is suggested that meteorological data is pre-disposed through strict quality control and homogenized. However, this data is doubted in the Discussion Section.

**Reply:** Thank you for your question. Indeed, the collected data from stations in the Altai Mountains is pre-disposed through strict quality control and homogenized. In the Discussion section, we doubted the fact that there has been a lack of consistency in the observation instrument and environments, such as vegetation changes, human grazing and relocation of stations because the Altai Mountains stretch over four countries (Kazakhstan, China, Mongolia and Russia).
